# Is trofinetide a future treatment for Rett syndrome? A comprehensive systematic review and meta-analysis of randomized controlled trials

**DOI:** 10.1186/s12916-024-03506-9

**Published:** 2024-07-18

**Authors:** Hazem E. Mohammed, Zeyad Bady, Mohamed E. Haseeb, Heba Aboeldahab, Wessam E. Sharaf-Eldin, Maha S. Zaki

**Affiliations:** 1 Faculty of Medicine, Assiut University, Assiut, Egypt; 2 Faculty of Medicine, Minia University, Minia, Egypt; 3 Medical Research Group of Egypt (MRGE), Negida Academy, Cairo, Egypt; 4 Clinical Research Department, El-Gomhoria General Hospital, MOHP, Alexandria, Egypt; 5 Biomedical Informatics and Medical Statistics Department, Medical Research Institute, Alexandria University, Alexandria, Egypt; 6 Medical Molecular Genetics Department, Human Genetics and Genome Research Institute, National Research Centre, Cairo, Egypt; 7 Clinical Genetics Department, Human Genetics and Genome Research Institute, National Research Centre, Cairo, Egypt; 8 Medical Genetics Department, Armed Forces College of Medicine (AFCM), Cairo, Egypt

**Keywords:** Rett syndrome, RTT, Trofinetide, Neurodevelopmental disorders, Systematic review, Meta-analysis

## Abstract

**Background:**

Rett syndrome (RTT) is a rare, life-threatening, genetic neurodevelopmental disorder. Treatment in RTT encounters many challenges. Trofinetide, a modified amino-terminal tripeptide of insulin-like growth factor 1, has demonstrated clinically promising results in RTT. In this study, trofinetide efficacy and safety in RTT are systematically reviewed and meta-analyzed.

**Methods:**

A systematic search of five electronic databases was conducted until January 2024. Review Manager 5.4 software was used for the analysis. The analysis was based on a weighted mean difference and standard error with a confidence interval (CI) of 95%, and a statistically significant *P*-value was considered if it was < 0.05. The study was registered on PROSPERO with registration number CRD42024499849. Quality of evidence was assessed using GRADE.

**Results:**

Three randomized controlled trials (RCTs) with 276 patients were included in the analysis. Trofinetide improved both caregiver outcomes and clinical scales by improving the Rett Syndrome Behavior Questionnaire (RSBQ) (mean difference (MD): − 3.46 points, 95% CI: − 5.63 to − 1.27, *P* = 0.0002) and Clinical Global Impression Scale–Improvement (CGI-I) (MD: − 0.35, 95% CI: − 0.51 to − 0.18, *P* < 0.0001), respectively. However, trofinetide neither improved the Caregiver Top 3 Concerns Visual Analog Scale nor the Rett Motor Behavioral Assessment. Regarding safety, trofinetide was significantly associated with vomiting compared to placebo (odds ratio (OR): 3.17, 95% CI: 1.57 to 6.43, *P* = 0.001). After solving heterogeneity, results showed a statistically significant incidence of diarrhea in the trofinetide (200 mg) group compared to placebo (OR: 18.51, 95% CI: 9.30 to 36.84, *P* ≤ 0.00001).

**Conclusions:**

Trofinetide demonstrated statistically significant improvements in CGI-I and RSBQ in pediatrics and adult patients with Rett. Side effects are limited to vomiting and diarrhea. Although diarrhea yielded an insignificant result in our analysis, it emerged as a cause for treatment discontinuation in the participating trials, and a statistically significant risk for diarrhea emerged when excluding the study using a lower dose of the drug, hence causing heterogeneity, in the meta-analysis. Given the diverse genetic landscape of RTT, future RCTs investigating correlations between RTT genotype and phenotypic improvements by trofinetide will be beneficial. RCTs encompassing male patients with larger and longer cohorts are recommended.

**Graphical Abstract:**

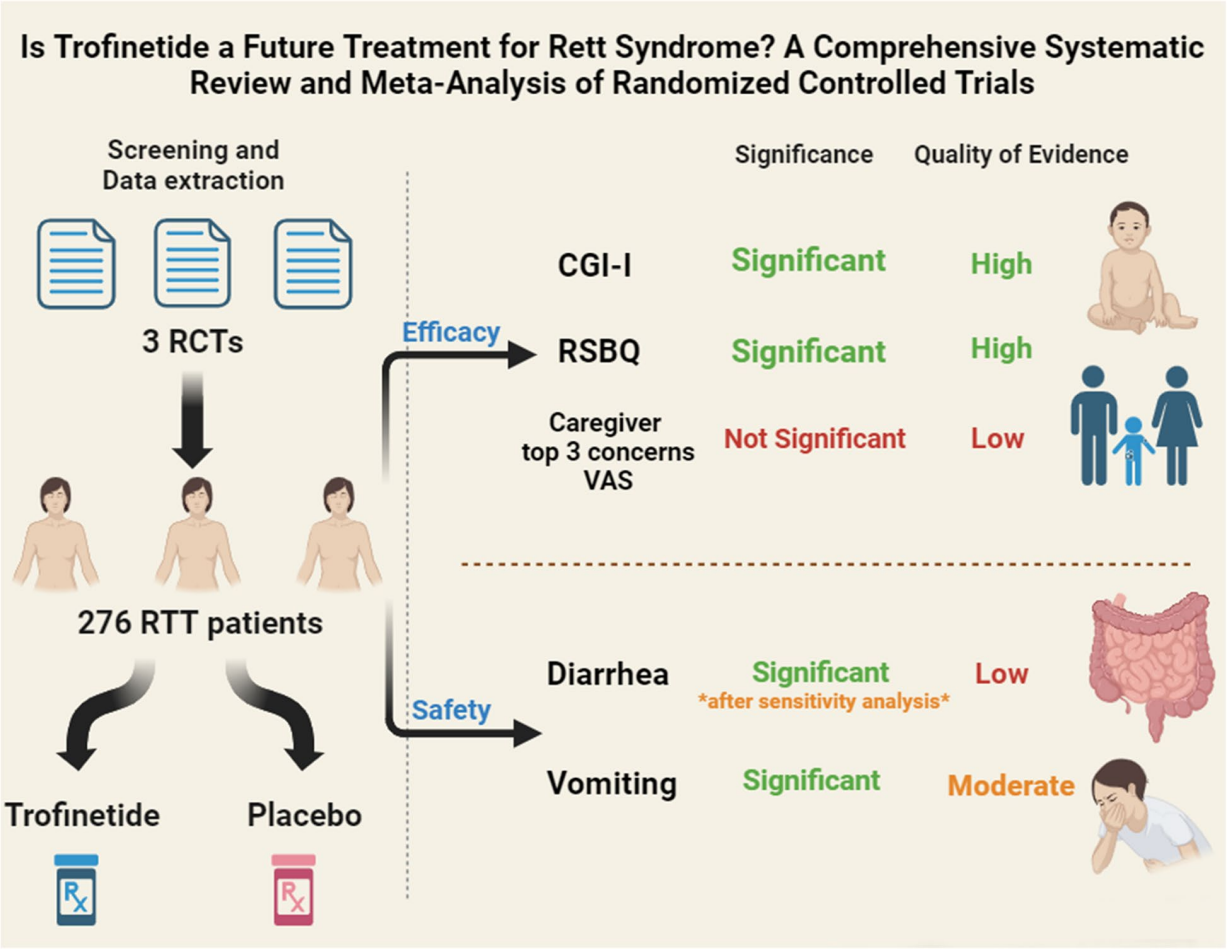

**Supplementary Information:**

The online version contains supplementary material available at 10.1186/s12916-024-03506-9.

## Background

Rett syndrome (RTT) is a rare neurodevelopmental disease that predominantly affects girls and is mainly caused by mutations in the methyl-CpG-binding protein 2 (*MECP2*) gene, responsible for the synthesis of the MeCP2 protein [1]. MeCP2 binds to methylated genomic DNA, which is crucial for various physiological functions, including normal neurological development [2]. Consequently, patients with RTT suffer from several neurological disruptions, including speech difficulty, motor dysfunction, hand stereotypies, abnormal gait, and epilepsy [1]. Besides neurological manifestations, RTT affects other systems, leading to abnormalities in growth, the gastrointestinal tract, breathing, and pubertal development [3]. The prevalence of RTT is nearly 5 to 10 cases per 100,000 females worldwide [4]. The cumulative incidence is 1.09 per 10,000 girls by the time they are 12 years old [1].

The primary management approaches to RTT involve providing symptomatic and supportive treatment, comprising behavioral, physical, occupational, and speech therapy, as well as medication to control seizures and other symptoms. Occupational therapy seeks to lessen stereotypic movements, enhance appropriate hand use, and facilitate daily activity performance. Also, physical and language therapies are required to improve mobility and social life [5]. Studies have assessed the effectiveness of specific drugs in controlling the symptoms of RTT. Naltrexone, an opioid antagonist, was evaluated as a potential treatment for periodic breathing. However, it was associated with significant deterioration in motor function and disorder progression [6]. The discovery of *MECP2* mutations and the possible significance of DNA methylation led to the implementation of a folate-betaine trial. Although parents reported improvements, no objective evidence was found [7].

Trofinetide (chemical name: glycyl-l-2-methylprolyl-l-glutamic acid), an analog of insulin-like growth factor-1 (IGF1), was considered a potential treatment for RTT. Phase II and III clinical trials, during which it showed improvement in various RTT symptoms at maximum dose [8]. Research conducted on mice with *MECP2* mutations showed that the amino-terminal tripeptide of insulin-like growth factor 1 (IGF-1), also known as glypromate, can significantly enhance physiological behavior and improve survival rates [9]. However, it had low bioavailability due to its rapid degradation [10, 11]. Trofinetide (brand name: Daybue), the synthetic analog of glypromate, which showed more resistance to degradation [12], has been approved by the US Food and Drug Administration (FDA) as a treatment for RTT in patients 2 years of age and older [13]. Various potential mechanisms of action have been suggested for trofinetide. The stimulation of synaptic maturation and function is one possible mode of action. It also restores dendritic morphology, neuronal signaling, and synaptic protein synthesis to normal, which are all essential for healthy neuronal function. In addition, it shields neurons from damage caused by oxidative stress via its antioxidant response [12, 14–17].

Trials conducted by Glaze et al. [18, 19] and Neul et al. [20] evaluated trofinetide’s efficacy, tolerability, and safety in RTT patients. Additionally, a study conducted by Neul et al. showed that trofinetide exhibited a significant change compared to placebo in the communication ability measuring scales, the caregiver-rated Communication and Symbolic Behavior Scales Developmental Profile™ Infant–Toddler Checklist (CSBS-DP-IT) Social Composite score and the Rett syndrome clinician rating of ability to communicate choices (RTT-COMC) [21]. Furthermore, an exposure–response (E-R) efficacy model by Darwish et al. [22] demonstrated that high trofinetide exposure improved the Rett Syndrome Behavior Questionnaire (RSBQ), CSBS-DP-IT, and RTT-COMC scores. Trofinetide was much better than placebo in reducing RSBQ total scores, with five to seven times greater reductions assuming target trofinetide with area under the concentration–time curve for the dosing interval 0 to 12 h (AUC_0–12_) values of 800–1200 μg·h/mL [22]. LILAC study by Percy et al. [23], a phase III open-label extension study of Neul et al. study [20], assessed the safety and efficacy of trofinetide after 40 weeks of treatment in 154 females with RTT, aged 5–21 years. Trofinetide treatment in this trial was found to sustain improvement in RTT symptoms as measured by several scales like RSBQ [23, 24].

In the present study, we aimed to provide class-one evidence demonstrating the efficacy and safety of trofinetide in RTT patients. We addressed several domains regarding RTT, including caregiver-completed assessments (Rett Syndrome Behavior Questionnaire (RSBQ), and Caregiver Top 3 Concerns), clinician-completed global assessment (Clinical Global Impression–Improvement (CGI-I)), and clinician-completed syndrome-specific assessments (Motor Behavior Assessment (MBA)). Furthermore, we aimed to provide insights into the safety profile of the drug. Besides, we investigated sources of heterogeneity whenever possible using sensitivity analysis and provided assessment of quality of evidence using GRADE.

## Methods

This systematic review and meta-analysis followed the criteria of the Preferred Reporting Items for Systematic Review and Meta-analysis (PRISMA) statement [25]. The protocol held a registration number of CRD42024499849 on PROSPERO.

### Eligibility criteria

We included studies meeting these criteria: (1) randomized controlled trials (RCTs); (2) studies including patients diagnosed with RTT; (3) the intervention group was trofinetide and the comparator was placebo; (4) English-language studies only. However, any studies meeting the following criteria: (1) observational studies, case reports, and conference abstracts; (2) studies with duplicated or overlapping populations; (3) uncontrolled studies; and (4) studies not written in English were excluded.

### Search strategy

Rigorous and comprehensive research was conducted from inception until January 2024 in Scopus, PubMed, WOS, Clinical trials.gov, and Cochrane Central Register of Controlled Trials (CENTRAL) databases. The search strategy comprised specific keywords and MeSH terms, including the following: “Trofinetide,” “Glycyl-l-2-methylprolyl-l-glutamic acid,” “RTT,” “Rett syndrome,” and “*MECP2.*” The search strategy used in the aforementioned databases is summarized in Additional file 1: Table S1.

### Study selection data extraction

After conducting the search strategy, two authors blindly performed studies screening on Rayyan online software [26]. We began initially with title-abstract screening and followed it with the screening of full text. A third reviewer was consulted to resolve any conflict between the two authors in the inclusion decision.

Two authors have extracted data independently on an online Excel sheet for easier access and connection between authors. The online sheet was divided into study characteristics, population baseline characteristics, and outcome measures data. Study characteristics included study name and year, sample size, design, duration of treatment, population, and key findings. Population baseline characteristics included sample size, age, body mass index (BMI) (kg/cm^2^), race, and ethnicity. Outcome measures involved:

1. Clinician-completed syndrome-specific global measures: Clinical Global Impression Scale–Improvement (CGI-I) is an assessment by the clinician of how much the patient’s illness severity has changed compared to baseline. A standard rubric specific to RTT was used in all participating studies, as described by Neul et al. [27]. A seven-point Likert scale is used, with a maximum score of seven, which indicates the worst stage of RTT.
2. Clinician-completed syndrome-specific measures: Rett Motor Behavioral Assessment (MBA): a scale composed of 34 items, captured on a four-point Likert scale, grouped into 3 subscales: behavior/social, orofacial/respiratory, and motor/physical signs.
3. Caregiver-completed assessments like (A) Rett Syndrome Behavior Questionnaires (RSBQ) total score: a forty-five-item questionnaire, thirty-eight of which are divided into eight domains/subscales that represent the fundamental characteristics of RTT: general mood, breathing problems, hand behaviors, repetitive face movements, body rocking and expressionless face, night-time behaviors, fear/anxiety, and walking/standing. (B) Caregiver Top 3 Concerns Total Visual Analog Scale: a Rett-syndrome-specific measure comprising three signs or symptoms that caregivers individually determined as their top concerns they would like the intervention to improve. The severity of each concern is scored using a 10-cm visual analog scale (VAS) using the methodology described in Glaze et al. [18] and Glaze et al. [19].

### Quality assessment

We evaluated the risk of bias using Cochrane Collaboration’s risk of bias tool (RoB), which involved seven domains: random sequence generation, allocation concealment, blinding of participants and personnel, blinding of outcome assessment, incomplete outcome data, selective reporting, and any other biases [28]. Studies were denoted as high risk, low risk, or unclear risk in each domain mentioned. A study was then judged based on the total risks in all domains. Any discrepancies were resolved by the opinion of a third reviewer.

### Statistical analysis

Review Manager 5.4 software (RevMan) was used for the statistical analysis [29]. We used RevMan to synthesize forest plots in both continuous and dichotomous outcomes. A random-effect model was considered in all outcomes due to the diversity of the genotypes of the investigated populations across the participating studies. The analysis was based on a weighted mean difference and standard error, with a confidence interval (CI) of 95%, and a statistically significant *P*-value was considered if it was < 0.05. Using the Higgins score (*I*^2^), the heterogeneity of the included studies was evaluated, I-square values ≥ 50% were indicative of high heterogeneity [30]. Adverse events were reported as the number of events per study arm and pooled as odds ratio (OR). A chi-square *P* value less than 0.1 was considered significant heterogeneity. Furthermore, sensitivity analysis was conducted to assess the heterogeneity and robustness of the results whenever possible.

### Quality of evidence

The level of certainty of the generated evidence was assessed using the Grading of Recommendations, Assessment, Development and Evaluation criteria (GRADE) [31, 32] by the GRADEpro Guideline Development Tool (GDT) online tool [33]. GRADE tool assesses the evidence and classifies it into four levels of certainty: very low, low, moderate, and high, taking into consideration the following domains of evaluation: risk of bias, inconsistency, indirect evidence, imprecision, publication bias, and other domains like dose–response effect and plausible confounding.

## Results

### Literature search

**Fig. 1:**
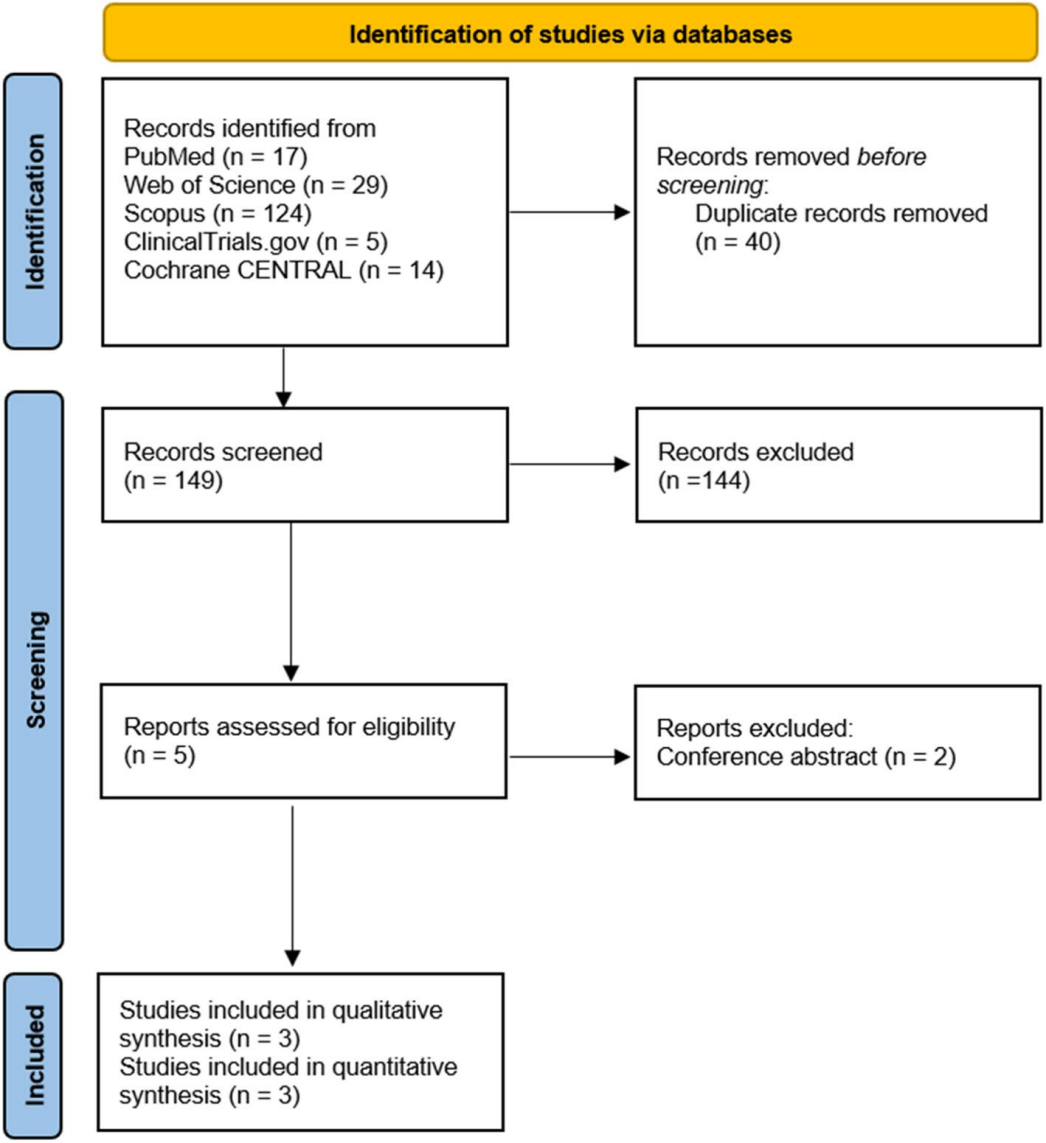
PRISMA flowchart of study selection

After we searched the three databases, we extracted 189 records. We identified 40 records as duplicates, which were removed. A total of 149 records underwent title-abstract screening, yielding five eligible records. Two records were eliminated as they did not meet the inclusion criteria. Finally, three RCTs [18–20] (*n* = 276) were included in both qualitative and quantitative analyses. The PRISMA flow diagram is shown in Fig. 1.

### Studies and population baseline characteristics

All three included studies are RCTs where patients were randomized to receive either trofinetide or placebo. Two studies, Glaze et al. [18, 19], were phase 2, while Neul et al. [20] study was phase 3. All remaining study characteristics, including sample size, duration of treatment, and key findings, are summarized in Table 1. Moreover, the characteristics of the studies’ population are represented in Table 2.

**Table 1:** Summary of the included study characteristics

**Study name and year**	**Neul et al. (2023)** [20]	**Glaze et al. (2019)** [19]	**Glaze et al. (2017)** [18]
Sample size	187	82	56
Design	Randomized double-blind, phase 3, placebo-controlled study	Exploratory phase 2, Multicenter, double-blind, placebo-controlled, parallel-group trial	Exploratory phase 2, Multicenter, double-blind, placebo-controlled, dose-escalation study
Population	187 females between 5 and 20 years of age with RTT, were assigned randomly to receive trofinetide (*n* = 93) or placebo (*n* = 94)	82 girls aged 5 to 15 years were diagnosed with RTT and had molecular evidence of a pathogenic MECP2 variant	56 female patients with RTT. All of them were included in the safety analysis, while the efficacy analysis included 55 of them
Intervention	Oral or via gastrostomy tube trofinetide	Oral or via gastrostomy tube trofinetide	Oral trofinetide
Dose	200 mg/ml	50, 100, or 200 mg/kg of trofinetide for 42 days. Another 20 participants were randomized to the placebo or 200 mg bid	35 mg/kg twice a day and 70 mg/kg twice a day
Comparator	Placebo	Placebo	Placebo
Study duration	Twelve weeks	Fifty-six days	Twenty-eight days for 70 mg/kg and 35 mg/kg cohorts and 14 days for another 35 mg/kg cohort
Outcome measures	CGI-I and RSBQ	MBA, CGI-I, Caregiver Top 3 Concerns Total VAS score, RSBQ, and RTT-DSC	MBA, CGI-I, Caregiver Top 3 Concerns Total VAS score, and ABC
Key findings	Trofinetide resulted in a significant improvement in both RSBQ and CGI-I compared to placebo, with *P* values equal to 0.0175 and 0.0030 respectively. The most common side effects were diarrhea and vomiting	Trofinetide at 200 mg/kg bid demonstrated significant clinical improvements in RSBQ, CGI-I, and RTT-DSC compared to placebo with *P* values equal to 0.042, 0.029, and 0.029 respectively. The most commonly reported adverse effects were diarrhea and vomiting	Trofinetide at a dose of 70 mg/kg showed efficacy compared to a placebo in MBA (*P* = 0.146), CGI-I (*P* = 0.164), and Caregiver Top 3 Concerns Total Visual Analog Scale (*P* = 0.076). The most common adverse effects were diarrhea, irritability, and somnolence

*RTT* Rett syndrome, *RSBQ* The Rett Syndrome Behavior Questionnaire, *CGI-I* Clinical Global Impression Scale–Improvement, *RTT-DSC* The Rett Syndrome-Clinician Domain Specific Concerns, *MBA* Rett Motor Behavioral Assessment, *ABC* Aberrant Behavior Checklist, *VAS* visual analog score, *MECP2* methyl CpG binding protein 2

**Table 2:** Baseline characteristics of study population

**Study names and groups**		**Neul et al. (2023)** [20]		**Glaze et al. (2019)** [19]				**Glaze et al. (2017)** [18]		
		Placebo	Trofinetide (200 mg/kg)	Placebo	Trofinetide (50 mg/kg)	Trofinetide (100 mg/kg)	Trofinetide (200 mg/kg)	Placebo	Trofinetide (35 mg/kg)	Trofinetide (70 mg/kg)
**Sample size**		94	93	24	15	16	27	20	18	17
**Age, years, mean (SD)**		10.9 (4.57)	11.00 (4.69)	9.38 (3.26)	10.06 (3.18)	10.81 (3.10)	9.23 (3.88)	27.41 (8.33)	23.74 (6.60)	24.52 (5.85)
**BMI, kg/cm**^**2**^**, mean (SD)**		N/A	N/A	16.00 (2.85)	16.50 (3.61)	17.70 (5.06)	16.31 (3.57)	21.54 (5.27)	23.45 (7.34)	20.5 (6.77)
**Race, number (%)**	**White**	90 (95.7%)	82 (88.2%)	22 (92%)	15 (100%)	15 (94%)	25 (93%)	19 (95%)	15 (83.3%)	15 (88%)
	**Asian**	1 (1.1%)	5 (5.4%)	1 (4%)	0 (0%)	0 (0%)	2 (7%)	0 (0%)	0 (0%)	1 (6%)
	**Black or African American**	1 (1.1%)	1 (1.1%)	0 (0%)	0 (0%)	1 (6%)	0 (0%)	1 (5%)	3 (16.7%)	1 (6%)
	**Other**	2 (2.1%)	5 (5.4%)	1 (4%)	0 (0%)	0 (0%)	0 (0%)	0 (0%)	0 (0%)	0 (0%)
**Ethnicity, Number (%)**	**Hispanic**	N/A	N/A	0 (0%)	1 (7%)	1 (6%)	6 (22%)	3 (15%)	0 (0%)	2 (12%)
	**Not Hispanic**	N/A	N/A	24 (100%)	14 (93%)	14 (88%)	21 (78%)	17 (85%)	18 (100%)	15 (88%)
**Age category, number (%)**	** ≤ 10**	52 (55.3%)	49 (52.7%)	15 (63%)	10 (67%)	10 (63%)	17 (63%)	N/A	N/A	N/A
	** > 10**	42 (44.7%)	44 (47.3%)	9 (38%)	5 (33%)	6 (38%)	10 (37%)	N/A	N/A	N/A

*N/A* not available, *SD* standard deviation

### Quality assessment

**Fig. 2:**
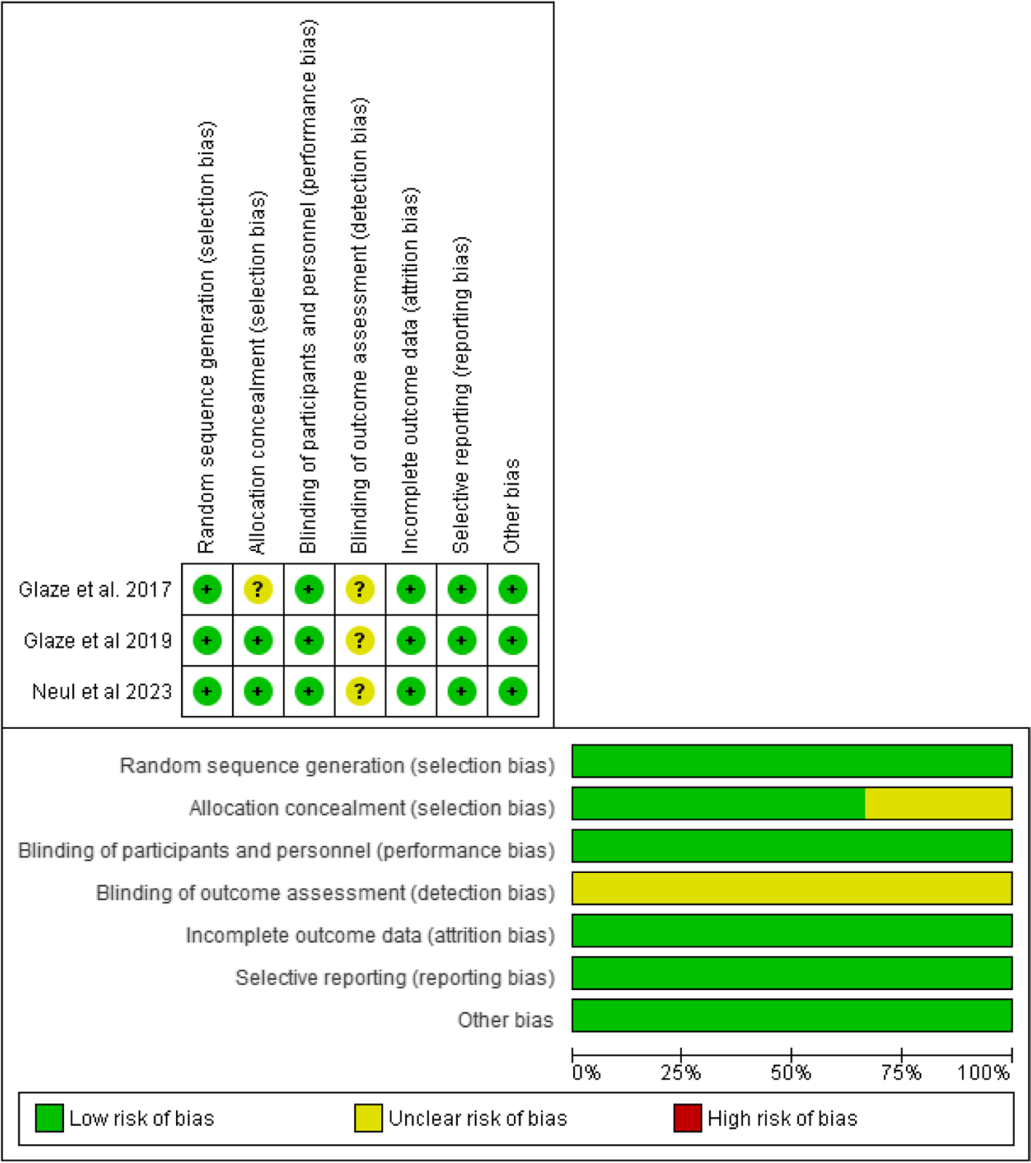
The risk of bias summary and risk of bias graph

According to Cochrane’s risk of bias assessment tool, the studies’ quality assessment was attributed to each and represented in both the graph and summary of the risk of bias as shown in Fig. 2.

### Efficacy of trofinetide

A. Clinician-completed syndrome-specific global measures (CGI-I)

**Fig. 3:**
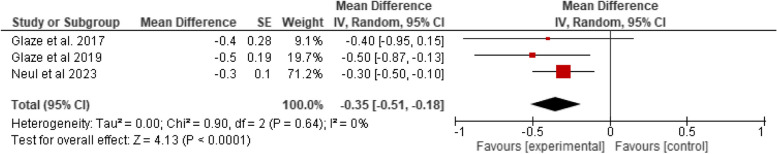
Comparison of trofinetide vs placebo in terms of CGI-I

Trofinetide exhibited a statistically significant reduction in CGI-I score compared to placebo (MD: − 0.35, 95% CI: − 0.51 to − 0.18, *P* < 0.0001), as shown in Fig. 3.

B. Clinician-completed syndrome-specific measures (MBA)

**Fig. 4:**
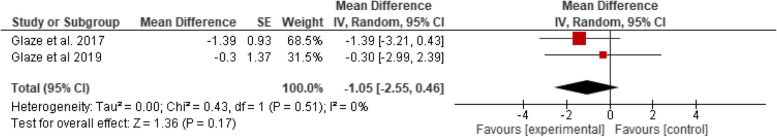
Comparison of trofinetide vs placebo in terms of MBA

The analysis showed no statistically significant difference between the drug and placebo in terms of MBA score (MD: − 1.05, 95% CI: − 2.55 to 0.46, *P* = 0.17), as shown in Fig. 4.

C. Caregiver-completed syndrome-specific measures (RSBQ and Caregiver Top 3 Concerns VAS)

**Fig. 5:**
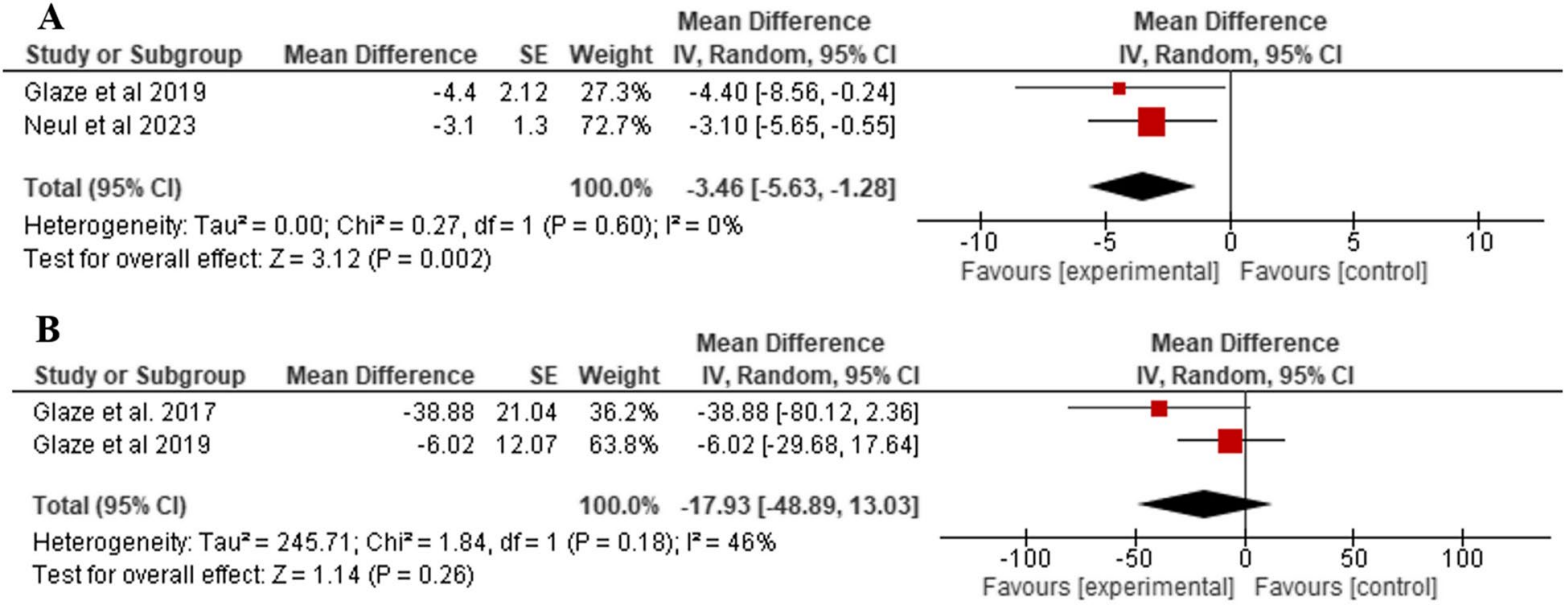
Comparison of trofinetide vs placebo in terms of **A** RSBQ and **B** Caregiver Top 3 Concerns VAS

Compared to placebo, the drug showed a statistically significant reduction regarding RSBQ total score (MD: − 3.46, 95% CI: − 5.63 to − 1.27, *P* = 0.0002), as shown in Fig. 5A. However, no statistically significant reduction was found between trofinetide and placebo with respect to the caregiver’s top 3 concerns VAS outcome (MD: − 17.93, 95% CI: − 48.89 to 13.03, *P* = 0.26), as shown in Fig. 5B.

### Safety of trofinetide

**Fig. 6:**
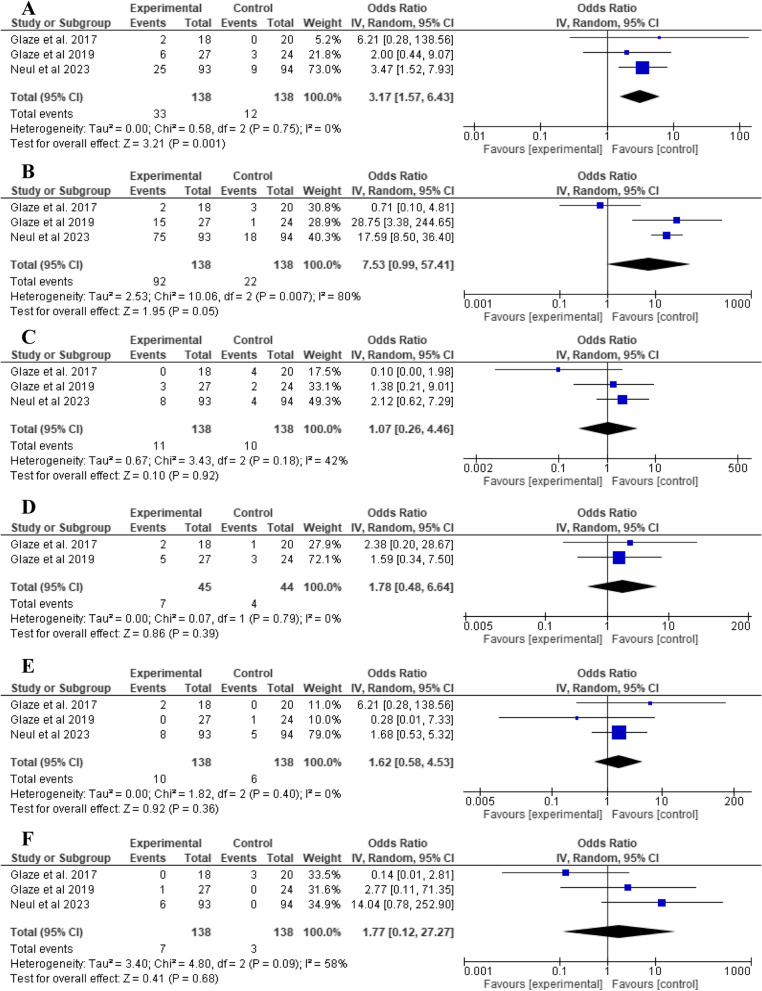
Comparison of trofinetide vs placebo in terms of **A** vomiting, **B** diarrhea, **C** pyrexia, **D** upper respiratory tract infections, **E** seizures, and **F** irritability

Trofinetide was significantly associated with a higher incidence of vomiting compared to placebo (OR: 3.17, 95% CI: 1.57 to 6.43, *P* = 0.001), as shown in Fig. 6A. The analysis revealed no significant difference between trofinetide and placebo with regard to diarrhea (OR: 7.53, 95% CI: 0.99 to 57.41, *P* = 0.05, Fig. 6B), pyrexia (OR: 1.07, 95% CI: 0.26 to 4.46, *P* = 0.92, Fig. 6C), upper respiratory tract infection (OR: 1.78, 95% CI: 0.48 to 6.64, *P* = 0.39, Fig. 6D), seizures (OR: 1.62, 95% CI: 0.58 to 4.53, *P* = 0.36, Fig. 6E), and irritability (OR: 1.77, 95% CI: 0.12 to 27.27, *P* = 0.68, Fig. 6F).

### Sensitivity analysis

**Fig. 7:**
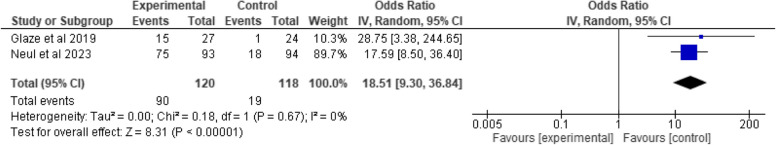
Comparison of trofinetide vs placebo in terms of diarrhea after removing Glaze et al. [18]

We conducted a sensitivity analysis for diarrhea outcome in order to investigate sources of heterogeneity and to test the stability and robustness of the result. The heterogeneity in diarrhea was resolved by removing Glaze et al. [18]. Remarkably, the pooled results showed a statistically significant incidence of diarrhea in the trofinetide group compared to placebo (OR: 18.51, 95% CI: 9.30 to 36.84, *P* ≤ 0.00001, Fig. 7).

### Quality of evidence

The quality of evidence regarding trofinetide efficacy and safety in the most important and relevant outcomes versus placebo was assessed using GRADE. Regarding efficacy, CGI-I and RSBQ were not downgraded at any level and yielded high-quality evidence. MBA was downgraded in the imprecision domain. Caregiver Top 3 Concerns VAS was downgraded in two domains: imprecision and inconsistency. Concerning safety, both vomiting and diarrhea were downgraded. The former was downgraded in the imprecision domain only, while the latter was downgraded in two domains: imprecision and inconsistency. A summary of the findings and a GRADE evaluation of the outcomes are represented in Table 3.

**Table 3:** Summary of findings and quality of evidence

Certainty assessment							Summary of findings				
**Outcomes/number of patients (number of studies)**	**Risk of bias**	**Inconsistency**	**Indirectness**	**Imprecision**	**Publication bias**	**Overall certainty of evidence**	**Study event rates (%)**		**Relative effect (95% CI)**	**Anticipated absolute effects**	
							**With placebo**	**With trofinetide**		**Risk with placebo**	**Risk difference with trofinetide**
**CGI-I**											
266 (3 RCTs)	Not serious	Not serious	Not serious	Not serious	None	⨁⨁⨁⨁ High	129	137	-	-	MD **0.35 points lower** (0.51 lower to 0.18 lower)
**MBA**											
79 (2 RCTs)	Not serious	Not serious	Not serious	Serious^**b**^	None	⨁⨁⨁◯ Moderate	35	44	-	-	MD **1.05 point lower** (2.55 lower to 0.46 higher)
**RSBQ**											
238 (2 RCTs)	Not serious	Not serious	Not serious	Not serious	None	⨁⨁⨁⨁ High	118	120	-	-	MD **3.46 point lower** (5.63 lower to 1.28 lower)
**Caregiver top 3 concerns visual analog scale**											
79 (2 RCTs)	Not serious	Serious^**a**^	Not serious	Serious^**b**^	None	⨁⨁◯◯ Low	35	44	-	-	MD **17.93 points lower** (48.89 lower to 13.03 higher)
**Diarrhea**											
276 (3 RCTs)	Not serious	Serious^**a**^	Not serious	Serious^**b**^	None	⨁⨁◯◯ Low	22/138 (15.9%)	92/138 (66.7%)	**OR 7.53** (0.99 to 57.41)	159 per 1000	**429 more per 1000** (from 1 fewer to 756 more)
**Vomiting**											
276 (3 RCTs)	Not serious	Not serious	Not serious	Serious^**b**^	None	⨁⨁⨁◯ Moderate	12/138 (8.7%)	33/138 (23.9%)	**OR 3.17** (1.57 to 6.43)	87 per 1000	**145 more per 1000** (from 43 to 293 more)

*RCT* randomized controlled trial, *CI* confidence interval, *MD* mean difference, *OR* odds ratio, *CGI-I* Clinical Global Impression Scale–Improvement, *MBA* Rett Motor Behavioral Assessment, *RSBQ* Rett Syndrome Behavior Questionnaires, *None* Not applicable because of the small number of included studies

^a^Wide variance of point estimates across studies

^b^Wide 95% confidence intervals, which include clinically important differences

## Discussion

The present meta-analysis provided class-one evidence about trofinetide efficacy and safety in RTT patients. It included three studies with 276 patients, aged 5–44 years, in the pooled analysis. Regarding the clinician-completed measures, trofinetide significantly improved RTT severity by reducing the CGI-I score by 0.35 points. Besides, the caregiver-completed assessments were improved by reducing the RSBQ total score by almost three and a half points. Both of the aforementioned outcomes are complementary and reflect functionally critical dimensions of RTT. As the most widely employed instrument in RTT investigations, the RSBQ is validated over a broad spectrum of ages (2–47 years) and has associations with functioning [34–36]. Furthermore, the CGI-I, which is also a widely used clinical scale in RTT trials, adds clinical context to a care-giver outcome like RSBQ [18, 19, 37–41]. It is noteworthy that the changes in CGI-I and RSBQ total scores in this meta-analysis are almost similar to the changes exhibited across all the participating trials. Given that all the participating trials had a Cohen’s *d* effect size for their CGI-I and RSBQ outcomes ranging from 0.4 to 0.6, this implies medium clinically meaningful improvements [42]. The most notable side effect of these improvements was vomiting (*P *= 0.001). Interestingly, diarrhea was not significant (*P* = 0.05).

It is worth mentioning that diarrhea was the most frequent and leading cause of treatment discontinuations, if present, among participants [19, 20]. However, it did not influence overall tolerability and resolved shortly after trofinetide cessation. Even a subset of caregivers who experienced diarrhea with their patients reported their willingness to continue treatment [43]. Although the diarrhea intensity exceeded the caregivers’ expectations, it was viewed as being difficult to manage. However, the caregivers reported some strategies to deal with diarrhea like increasing dietary fiber, being prepared for cleanup, and adjusting the drug dose [43]. Although lowering the dose improved diarrhea in some candidates, a post-hoc analysis of the Neul et al. study (LAVENDER) revealed that the starting dose didn’t significantly affect the incidence of diarrhea. The rates were 69.2%, 88.0%, 83.3%, and 83.3% for initial doses of 150 to < 250, 250 to < 300, 300 to < 350, and 350 to < 500 mg/kg administered twice daily, respectively [44]. However, it is important to note that the aforementioned doses are relatively large and were a source of heterogeneity in our meta-analysis. Although diarrhea was statistically insignificant in our analysis (*P* = 0.05), sensitivity analysis by removing Glaze et al. [18] yielded a statistically significant risk for developing diarrhea (OR: 18.51, 95% CI: 9.3 to 36.84, *P* = 0.001). Glaze et al. [18] used a lower dose of 70 mg/kg compared to 200 mg/kg in Glaze et al. [19] and Neul et al. [20]. The dosing effect might also explain the low prevalence of diarrhea in other trials using trofinetide for fragile X syndrome that used doses of 35 and 70 mg/kg daily [45]. In addition to dosing, RTT is associated with constipation [46, 47]. Constipation is managed in RTT using different medications [48, 49] as reported in Neul et al. study [20] where about 60% of trofinetide group was taking drugs to treat constipation, which might be considered, in theory, a confounding element. It is unclear whether incidence of diarrhea was similar between RTT patients on or off drugs to treat constipation. However, it appears that RTT increases the susceptibility to trofinetide-induced diarrhea; in a phase 1 study, 41 healthy adults were administered trofinetide approximately 160 mg/kg once daily, and no treatment-emergent adverse events (TEAEs) related to diarrhea were reported [50]. Therefore, it is recommended to stop or reduce all drugs to treat constipation upon starting trofinetide and consider an individualized approach for each patient [44].

An individualized approach is crucial in RTT treatment because a pivotal contributor to the phenotype is the location of the mutation on the X chromosome. The mutation location contributes to various degrees of X chromosome inactivation and skewing [51, 52]. The phenotype severity of RTT can also be correlated with the genotype. Patients who carried either missense variants or late truncating variants seem to have a milder phenotype. For instance, a study reported that patients with the R133C variant exhibited a less severe phenotype than those with the R168X variant. Moreover, the latter was associated with a more severe phenotype than R294X variant-bearing patients [53]. As a result, Glaze et al. [18] randomized participants according to loci of the *MECP2* variant, proximal to R294X versus R294X and distally, creating a similar distribution of variants across the experimented cohorts. Nevertheless, none of the involved clinical trials provided any information regarding the efficacy of trofinetide according to the different types of variants. As a consequence, it is quite challenging to determine any correlation between trofinetide and particular variants.

In the majority of participants, trofinetide positive effects were diminished after cessation of the drug. This might entail longer-term treatment to investigate such sequelae and substantiate increased clinical benefits with longer durations. Furthermore, the Glaze et al. [19] study showed more manifest improvements than that of Glaze et al. [18]. These improvements could be attributed to some clinically crucial factors. First and foremost, younger age was clearer in Glaze et al. [19] with a mean age of nine compared to 24 in Glaze et al. [18] (refer to baseline characteristics table). Aging has been linked to diminished neuroplasticity [54–56], and the lack of a functional *MECP2* protein—the most common cause of RTT—prematurely closes a window of neuronal plasticity in a brain region involved in social memory. Since trofinetide is believed to enhance synaptic functions and restore synaptic structure [12, 14, 15], it is likely to lead to more significant improvements if administered at a younger age. Second, drug doses were higher in the Glaze et al. (2019) study [19], corresponding to increased drug exposure. Finally, the longer duration—42 days compared to 28 days—might have contributed to the observed increased improvements. Besides, heterogeneity was evident in our pooled analysis of diarrhea and irritability (*I*^2^ = 80%, *P* = 0.007 and *I*^2^ = 58%, *P* = 0.09), respectively. We assume that the aforementioned differences regarding drug doses (i.e., 70 mg in Glaze et al. [18] compared to 200 mg in the others), different treatment durations, altered patients’ tolerability to the drug, administration of drugs to treat constipation, and a wide spectrum of genotype variations might account for the observed heterogeneity.

Males diagnosed with RTT were not enrolled in any of the clinical trials conducted until this time. *MECP2* variants have been believed for a long time to be lethal in males; however, such cases were identified with a wide range spectrum of phenotypes, and nine variants that have not been seen in females beforehand are reported in males. Eventually, this led to the introduction a new entity called “Male RTT Encephalopathy” [57]. Moreover, males exhibit *MECP2* duplication syndrome more frequently than RTT, which both have many clinical features in common [58]. The aforementioned aspects highlight the profound difference in RTT diagnosis between males and females accounting for the plausible exclusion of males from these clinical trials.

This study stands out at some points, including conducting a sensitivity analysis, which revealed that lower doses were associated with a lower incidence of diarrhea. Furthermore, we performed a GRADE assessment to evaluate the quality of the evidence, providing a more rigorous and structured appraisal of the evidence. Our study provided a detailed discussion on the incidence of diarrhea in RTT patients in the context of trofinetide treatment, aiming for a clearer understanding. The present study provided quantitative and qualitative evidence on the efficacy and safety of trofinetide on a wide spectrum of RTT patients, ranging from 5 to 44 years old. However, the optimism of this study must be titrated by the lack of assessing the safety and efficacy of trofinetide in the long term. Moreover, the analyzed cohort is small, which is understandable given the rarity of the investigated disease. Besides, males suffering from RTT were not enrolled in any of the participating studies due to phenotype variability in males. Moreover, the youngest age investigated in the participating studies was 5 years. We believe that earlier administration of the drug may highlight new aspects about the efficacy and safety of the drug. Another limitation is the absence of biomarkers determining responders to trofinetide treatment. Therefore, we recommend that future studies should focus on conducting more RCTs assessing both younger age groups, (i.e., below 5 years of age), and longer-duration treatments to evaluate their effect on treatment efficacy. In addition, they have to consider genotype–phenotype relationships by dividing participants into subgroups based on their mutational type and evaluate the drug efficacy between different mutations. RCTs on males, exclusively, assessing a wide spectrum of mutations are also recommended.

## Conclusions

In conclusion, this meta-analysis of trofinetide demonstrated statistically significant, moderate clinical improvement in complementary scales of RTT like CGI-I and RSBQ. The safety profile of trofinetide revealed a significant increase in vomiting compared to placebo. Although diarrhea yielded an insignificant result in our analysis, it emerged as a cause for treatment discontinuation in the participating trials, and a statistically significant risk for diarrhea emerged when excluding the study using a lower dose of the drug, hence causing heterogeneity, in the meta-analysis. We underscored the importance of considering genotype–phenotype correlations in evaluating trofinetide’s efficacy, especially given the diverse genetic landscape of RTT. Furthermore, younger ages, longer durations, and male-limited RCTs are required.

### Supplementary Information

Additional file 1: Table S1. Search strategy utilized in all databases.

## Data Availability

All data generated or analyzed during this study are included in this published article [and its supplementary information files].

## Abbreviations

ABC: Aberrant Behavior Checklist
CGI-I: Clinical Global Impression Scale–Improvement
CI: Confidence interval
MBA: Rett Motor Behavioral Assessment
MD: Mean difference
MECP2: Methyl CpG binding protein 2
OR: Odds ratio
PRISMA: Preferred Reporting Items for Systematic Reviews and Meta-Analyses
RCTs: Randomized controlled trials
RevMan: Review Manager software
RoB: Cochrane risk-of-bias tool for randomized trials
RSBQ: The Rett Syndrome Behavior Questionnaires
RTT: Rett syndrome
VAS: Visual analog score
