# Supplementary material for: Is trofinetide a future treatment for Rett syndrome? A comprehensive systematic review and meta-analysis of randomized controlled trials
Source: BMC Med. 2024 Jul 18;22:299. doi: 10.1186/s12916-024-03506-9 (PMC11256568; doi:10.1186/s12916-024-03506-9)
Supplement: Supplementary file 1 — Additional file 1: Table S1. Search strategy utilized in all databases. [file 12916_2024_3506_MOESM1_ESM.docx]

**Is Trofinetide a Future Treatment for Rett Syndrome? A Comprehensive Systematic Review and Meta-Analysis of Randomized Controlled Trials**

Hazem E. Mohammed^1^**^*^**, Zeyad Bady^1^**^*^**, Mohamed E. Haseeb^2^, Heba Aboeldahab^3,4.5^, Wessam E. Sharaf-Eldin^6^, Maha S. Zaki^7,8^ **^#^**

***Affiliations***

*^1^ Faculty of Medicine, Assiut University, Assiut, Egypt*

*^2^Faculty of Medicine, Minia University, Minia, Egypt*

*^3^ Medical Research Group of Egypt (MRGE), Negida Academy, Cairo, Egypt*

*^4^ Clinical Research Department, El-Gomhoria General Hospital, MOHP, Alexandria, Egypt.*

*^5^ Biomedical Informatics and Medical Statistics Department, Medical Research Institute, Alexandria University, Alexandria, Egypt*

*^6^ Medical Molecular Genetics Department, Human Genetics and Genome Research Institute, National Research Centre, Cairo, Egypt*

*^7^ Clinical Genetics Department, Human Genetics and Genome Research Institute, National Research Centre, Cairo, Egypt*

*^8^ Medical Genetics Department, Armed Forces College of Medicine (AFCM), Cairo, Egypt*

***^*^These two authors contributed equally to this work and designated as co-first authors.***

**^#^*Corresponding author*: Maha S Zaki**

E-mail: dr_mahazaki@yahoo.com

Phone number: +201060633727

ORCID: https://orcid.org/0000-0001-7840-0002

Address: National Research Centre, Eltahrir Street, Cairo, 12622, Egypt

**Authors’ email addresses**

Hazem E. Mohammed: [hazemeslam25@gmail.com](mailto:hazemeslam25@gmail.com)

Zeyad Bady: [zeyadbady02@gmail.com](mailto:zeyadbady02@gmail.com)

Mohamed E. Haseeb: [mohamedemad41345@gmail.com](mailto:mohamedemad41345@gmail.com)

Heba Aboeldahab: [Hebaaboeldahab9@gmail.com](mailto:Hebaaboeldahab9@gmail.com)

Wessam E. Sharaf-Eldin: [wessam_sharafeldin@yahoo.com](mailto:wessam_sharafeldin@yahoo.com)

Maha S. Zaki: [dr_mahazaki@yahoo.com](mailto:dr_mahazaki@yahoo.com)

**Table S1. Search strategy**

| **Domain** | (RTT OR (Rett syndrome) OR "Rett disorder" OR "Rett’s disorder" OR "Rett syndrome" OR "Autism-dementia-ataxia-loss of purposeful hand use syndrome" OR MECP2 OR "MECP2 protein") |
| --- | --- |
| **Determinant** | (trofinetide OR daybue OR "glycyl-l-2-methylprolyl-l-glutamic acid" OR NNZ-2566) |
